# Mindfulness, Acceptance and Catastrophizing in Chronic Pain

**DOI:** 10.1371/journal.pone.0087445

**Published:** 2014-01-29

**Authors:** Maaike J. de Boer, Hannemike E. Steinhagen, Gerbrig J. Versteegen, Michel M. R. F. Struys, Robbert Sanderman

**Affiliations:** 1 Pain Center, Department of Anesthesiology, University of Groningen, University Medical Center Groningen, Groningen, The Netherlands; 2 Health Psychology Section, Department of Health Sciences, University of Groningen, University Medical Center Groningen, Groningen, The Netherlands; The University of Texas at Austin, United States of America

## Abstract

**Objectives:**

Catastrophizing is often the primary target of the cognitive-behavioral treatment of chronic pain. Recent literature on acceptance and commitment therapy (ACT) suggests an important role in the pain experience for the concepts mindfulness and acceptance. The aim of this study is to examine the influence of mindfulness and general psychological acceptance on pain-related catastrophizing in patients with chronic pain.

**Methods:**

A cross-sectional survey was conducted, including 87 chronic pain patients from an academic outpatient pain center.

**Results:**

The results show that general psychological acceptance (measured with the AAQ-II) is a strong predictor of pain-related catastrophizing, independent of gender, age and pain intensity. Mindfulness (measured with the MAAS) did not predict levels of pain-related catastrophizing.

**Discussion:**

Acceptance of psychological experiences outside of pain itself is related to catastrophizing. Thus, acceptance seems to play a role in the pain experience and should be part of the treatment of chronic pain. The focus of the ACT treatment of chronic pain does not necessarily have to be on acceptance of pain per se, but may be aimed at acceptance of unwanted experiences in general. Mindfulness in the sense of “acting with awareness” is however not related to catastrophizing. Based on our research findings in comparisons with those of other authors, we recommend a broader conceptualization of mindfulness and the use of a multifaceted questionnaire for mindfulness instead of the unidimensional MAAS.

## Introduction

Catastrophizing about pain is defined as an exaggerated negative “mental set” regarding actual or anticipated pain experiences [1]. Catastrophizing is related to higher levels of pain and suffering [2] and to an increased need for medical advice, higher use of over-the-counter medicine and increased disability [1]. Catastrophizing is often the primary target of cognitive-behavioral treatment (CBT), which has become the treatment of choice for chronic pain over the past decades. In recent years, a new form of behavior therapy has emerged, with an emphasis on contextual and experiential change strategies. This third generation behavior therapy is called acceptance and commitment therapy (ACT) [3] and is increasingly used in the treatment of chronic pain [4]. ACT focuses in particular on the concepts of acceptance, experiential avoidance and mindfulness [5].

Acceptance and experiential avoidance are two extremes of the same construct. Thus, a high level of acceptance means a low level of experiential avoidance. In the chronic pain literature, a distinction is made between general psychological acceptance (acceptance of undesirable experiences) and acceptance of chronic pain [6]. Thus, acceptance of pain is a specific form of general psychological acceptance. In chronic pain patients, acceptance of pain is associated with lower pain intensity, less pain-related anxiety and avoidance, less depression, less physical and psychosocial disability, more daily uptime and better work status [7]. General psychological acceptance is shown to be a significant predictor of psychological functioning in various clinical and non-clinical samples [8] and in a sample of patients with chronic pain [6]. According to McCracken and Zhao-O'Brien [6], general psychological acceptance plays a unique role in the chronic pain experience, beyond similar processes such as acceptance of pain and mindfulness.

Mindfulness is another key concept in the ACT model. Bishop [9] defines mindfulness as “a state of being aware of and focusing on the present moment”. One accepts the present moment without evaluating thoughts or emotional reactions to the situation. In chronic pain patients, mindfulness accounts for a significant amount of variance in measures of depression, pain-related anxiety and physical, psychosocial and other types of disability [10]. In addition, Schutze et al. [11] concluded on the basis of a cross-sectional study that mindfulness is a unique predictor of pain-related catastrophizing.

Current literature on chronic pain suggests an important role for concepts from the “third generation cognitive behavioral approach” like mindfulness and acceptance. The aim of the present study was to examine levels of mindfulness and general psychological acceptance in a sample of chronic pain patients and to investigate the relationship between both mindfulness and general psychological acceptance and pain-related catastrophizing in these patients. We hypothesized that mindfulness as well as acceptance are significant predictors of pain-related catastrophizing.

## Methods

### Subjects and procedure

All patients who were refered to the Pain Center of the University Medical Center Groningen in the Netherlands for interdisciplinairy pain treatment between November 2010 and April 2011 were asked to participate in the study. Data (Numeric Rating Scale and Pain Catastrophizing Scale) for the current study were partly collected in the course of standard medical care. At the time of their first visit to the Pain Center, patients were asked to fill in two additional questionnaires (Mindful Attention Awareness Scale and Acceptance and Action Questionnaire-II). Participation in the study was anonymous and voluntary.

Data from 89 chronic pain patients were collected. The sample consisted of 34 (37.8%) men and 55 (61.1%) women, with a mean age of 51.33 (SD  = 15.54, range 20–92). Of the participants, 62.9% were married, 10.1% were divorced, 13.5% were living together unmarried, 11.2% were single and 2.2% were widowed.

The UMCG Institutional Ethics Committee waived the requirement of approval. No WMO (Wet Medisch-wetenschappelijk onderzoek met mensen/Medical Research with Human Subjects Act) registration was necessary for this study in the Netherlands.

### Questionnaires

Subjects were asked to complete the following self-report questionnaires:

### Pain Catastrophizing Scale (PCS)

The Pain Catastrophizing Scale (PCS) is a 13-item self-report measure developed by Sullivan et al. [2] for use in both clinical and non-clinical populations. Participants are asked to reflect on a painful experience (“When I'm in pain…”) and to indicate on a 5-point scale the degree to which they experienced various thoughts and feelings. The PCS yields a total score, indicating the degree of pain-related catastrophizing. Next to this total score, three subscales can be calculated: magnification, rumination and helplessness. In the authorized Dutch version of the PCS, the three factor structure has been confirmed across different pain patient samples and a non-clinical sample [12]. The Dutch version of the PCS has a high internal consistency (Cronbach's alpha varies between 0.85 en 0.91 [13]) and the PCS has a good test-retest reliability (*r* = 0.75 over a period of six weeks and *r* = 0.70 over a period of ten weeks for the English version [2]). For the Dutch version of the PCS, norms are available for chronic pain patients (divided into chronic back pain patients and fibromyalgia patients) and healthy subjects (students, divided by gender) [13]. In our study, the PCS was part of the questionnaire booklet patients filled in prior to their first visit to the Pain Center as part of standard medical care.

### Mindful Attention Awareness Scale (MAAS)

The Mindful Attention Awareness Scale (MAAS) is a 15-item self-report measure developed by Brown and Ryan [14] measuring the frequency of everyday mindfulness experiences. The authors state that the MAAS measures a unique quality of conscienceness that is related to a variety of well-being constructs. Mindfulness as measured with the MAAS is a one-dimensional construct [14], [15]. The questionnaire yields a total score which is the mean of the 15 itemscores. A higher score indicates a higher level of mindfulness. Schroevers et al. [16] developed an authorized Dutch version of the MAAS. The Dutch version has a high internal consistency (Cronbach's alpha varies between 0.81 and 0.87) and the one-factor structure was confirmed in the Dutch version [16]. Participants completed the MAAS during their first visit to the Pain Center.

### Acceptance and Action Questionnaire-II (AAQ-II)

The Acceptance and Action Questionnaire was developed by Hayes in 2004 as a measure of experiential avoidance/acceptance [17]. Recently, an adapted version of the questionnaire was developed, i.e. the Acceptance and Action Questionnaire-II [18]. The AAQ-II appears to measure the same construct as the original AAQ with better psychometric consistency. The AAQ-II is sometimes referred to as a measure of psychological flexibility.

Jacobs et al. [19] translated the 10-item AAQ-II into Dutch. This authorized Dutch version has a high internal consistency (Cronbach's alpha  = 0.89) and the one-factor structure of the English version [6] was confirmed in the Dutch version. The AAQ-II yields a total score with a minimum of 10 and a maximum of 70. A high score on the AAQ-II indicates a high level of acceptance/psychological flexibility and thus a low level of experiential avoidance/psychological inflexibility. Participants completed the AAQ-II during their first visit to the Pain Center.

Next to these questionnaires, some questions regarding demographics, pain complaints, previous specialist consultation and medication use were asked. Also, pain intensity was measured with a Numeric Rating Scale (NRS). This NRS was accompanied by the following question: “How much pain did you experience during the last two days?”. The range of the NRS was 0 (no pain) to 10 (worst pain imaginable).

### Statistical analysis

Data were analysed with SPSS 18.0 using descriptive statistics, correlations and stepwise multiple linear regression analyses. In tests of statistical significance, the significance level was set at *p*≤0.05 (two-tailed). The central limit theorem justifies the use of parametric tests.

In order to compare the scores of our sample to samples from the literature, one-sample *t*-tests were used and in order to investigate the relationship between mindfulness, acceptance and catastrophizing, correlational coefficients (Pearson's *r*) were calculated. In order to describe the relationship between both mindfulness and acceptance and catastrophizing, a stepwise multiple linear regression analysis was conducted with mindfulness and acceptance as the independent variables and catastrophizing as the dependent variable. In the first step of the multiple linear regression analysis, age, sex and pain intensity were added. In the second step mindfulness was added to the analysis as predictor variable and in the third step acceptance was added.

We also conducted a moderated linear regression analysis to investigate whether the relationship between mindfulness and catastrophizing differed for different levels of acceptance. Following the procedure described by Frazier et al. [20], variables were standardized in order to reduce problems associated with multicollinearity among the variables. Next, the product term “mindfulness x acceptance” was calculated representing the interaction between mindfulness and acceptance. A stepwise multiple linear regression analysis with mindfulness, acceptance and the interaction term predicting catastrophizing was conducted.

## Results

### Levels of mindfulness, acceptance and catastrophizing

The mean scores of the participants on the Pain Catastrophizing Scale (catastrophizing), Mindful Attention Awareness Scale (mindfulness), Acceptance and Action Questionnaire-II (acceptance) and mean NRS pain scores are shown in Table 1.

**Table 1:** Means, standard deviations and range of measures used in the present study.

	N	Mean	SD	Min	Max
NRS pain	86	7.45	1.68	2	10
PCS	85	22.41	12.96	0	48
MAAS	88	4.20	0.86	2	6
AAQ-II	88	50.33	10.71	25	69

NRS  =  Numeric Rating Scale; PCS  =  Pain Catastrophizing Scale; MAAS  =  Mindful Attention Awareness Scale; AAQ-II  =  Acceptance and Action Questionnaire-II.

The mean experienced pain intensity on a NRS scale from 1 to 10 was 7.45 (SD  = 1.68, range 2–10), indicating a high pain severity. In order to relate the scores on the various questionnaires of our sample to other (pain) groups and thus gain insight into the meaning of the scale scores, we compared the scores to various samples and normgroups from previous studies using one-sample *t*-tests. The PCS scores were comparable to those in pain patient samples from other studies (pain outpatients sample of Osman et al. [21]: *t* (84) = 0.111, *p* = 0.912; chronic low back pain sample of Van Damme et al. [12]: *t* (84) = 0.296, *p* = 0.768; fibromyalgia sample of Van Damme et al. [12]: *t* (84) = −1.710, *p* = 0.091). However, the PCS scores in our sample were lower than those in a chronic pain patient sample from Schutze et al. [11] (*t* (84) = −2.721, *p* = 0.008).

When comparing the MAAS scores from our sample to previous research, we found that the scores in our sample were higher than Schutze et al.'s [11] chronic pain patient sample (*t* (88) = 4.324, *p*<0.001), indicating a higher level of mindfulness in our sample. In comparison to samples from the general population, our participants reported higher levels of mindfulness than Brown and Ryan's [14] sample (*t* (88) = 2.555, *p* = 0.012), but lower levels of mindfulness than Carlson and Brown's [15] sample (*t* (88) = −2.751, *p* = 0.007). We also compared our sample to the study by Schroevers et al. [16], who describe two samples from the general population. Our sample reported higher levels of mindfulness than their first sample (*t* (88) = 6.092, *p*<0.001), but comparable levels of mindfulness to their second sample (*t* (88) = 1.339, *p* = 0.184).

The AAQ-II scores of our sample were comparable to the scores of the general population sample from Jacobs et al. [19] (*t* (87) = −1.415, *p* = 0.161), but higher than their clinical sample from two psychiatric clinics (*t* (87) = 9.377, *p*<0.001), indicating a higher level of acceptance in our sample. The AAQ-II scores of our sample were also significantly higher than those of a sample of chronic pain patients of an interdisciplinairy pain clinic (*t* (87) = 10.356, *p*<0.001) [6].

### Relationships between mindfulness, acceptance and catastrophizing

The correlation coefficient between mindfulness and acceptance was quite strong, i.e. *r* (85) = 0.52, *p*<0.001 (Table 2). Furthermore, acceptance was significantly correlated with pain-related catastrophizing (*r* (82) = −0.42, *p*<0.001), higher levels of acceptance being related to lower levels of pain-related catastrophizing. Mindfulness showed no significant correlation with pain-related catastrophizing. The experienced pain intensity (as measured with a NRS-scale) was significantly correlated to pain-related catastrophizing (*r* (82) = 0.40, *p*<0.001), but not to mindfulness or acceptance.

**Table 2:** Correlations (Pearson's *r*) among pain intensity, catastrophizing, mindfulness and acceptance.

	NRS pain	PCS	MAAS
NRS pain	—		
PCS	0.40**	—	
MAAS	0.13	−0.14	—
AAQ-II	−0.02	−0.42**	0.52**

** *p*<0.001; NRS  =  Numeric Rating Scale; PCS  =  Pain Catastrophizing Scale; MAAS  =  Mindful Attention Awareness Scale; AAQ-II  =  Acceptance and Action.

Questionnaire-II.

### The influence of mindfulness and acceptance on catastrophizing

To examine whether levels of mindfulness and levels of acceptance predicted levels of pain-related catastrophizing in our chronic pain sample, a stepwise multiple linear regression analysis was used. In the first step of the regression analysis, age, sex and pain intensity were used. The analysis revealed that pain intensity was a significant predictor of pain-related catastrophizing in step 1 (Table 3). In step 2, mindfulness was added to the regression model. In this model mindfulness was not a significant predictor. With the adding of mindfulness, *R^2^* increased with only 0.04 (Table 4). In step 3, acceptance was added to the model. Acceptance was a significant predictor of pain-related catastrophizing. With the adding of acceptance, *R^2^* increased with 0.12. Thus, acceptance explained an additional 12% of the variance in pain-related catastrophizing over and above gender, age, pain intensity and mindfulness. The final model explained a significant proportion of variance in PCS scores (*R^2^* = 0.33; *F* (5, 77) = 7.59, p<0.001).

**Table 3:** Results of multiple linear regression analysis predicting PCS-scores (*n* = 81).

Model	Variables	*B*	*SE (B)*	*β*	*t*	*p*
1	Sex	−1.32	2.72	−0.05	−0.505	0.615
	Age	0.12	0.09	0.14	1.362	0.177
	Pain intensity (NRS)	3.11	0.80	0.40	3.888	<0.001**
2	Sex	−1.43	2.68	−0.06	−0.533	0.596
	Age	0.13	0.09	0.16	1.571	0.120
	Pain intensity (NRS)	3.34	0.80	0.43	4.190	<0.001**
	Mindfulness (MAAS)	−2.96	1.58	−0.19	−1.871	0.065
3	Sex	−0.29	2.51	−0.01	−0.116	0.908
	Age	0.09	0.08	0.11	1.109	0.271
	Pain intensity (NRS)	2.89	0.75	0.37	3.846	<0.001**
	Mindfulness (MAAS)	0.57	1.76	0.04	0.325	0.746
	Acceptance (AAQ-II)	−0.49	0.13	−0.41	−3.642	<0.001**

** *p*<0.001.

**Table 4:** Results of multiple linear regression analysis predicting PCS-scores (*n* = 81).

Model	*F*	*p*	*R^2^*	*ΔR^2^*
1	5.754	0.001*	0.18	0.18*
2	5.328	0.001*	0.22	0.04
3	7.585	<0.001**	0.33	0.12**

* *p*<0.05;

** *p*<0.001.

The finding that mindfulness was not a significant predictor of pain-related catastrophizing is contrary to our expectation. We conducted an additional moderator analysis to investigate whether this unexpeced result could be explained by a moderating effect of acceptance on the relationship between mindfulness and catastrophizing using the procedure described by Frazier et al. [20].

First, variables were standarized in order to reduce problems associated with multicollinearity among the variables. Next, we conducted a stepwise multiple linear regression analysis with mindfulness and acceptance added in the first step and the addition of the product term “mindfulness x acceptance” in the second step of the analysis. The results showed that the interaction between mindfulness and acceptance added no incremental variance (*R^2^* = 0.20) to pain-related catastrophizing (*B* = 0.00, *p* = 0.976) (Table 5). Acceptance did not significantly moderate the relationship between mindfulness and catastrophizing. Thus, the finding that mindfulness was not a significant predictor of pain-related catastrophizing was not due to a moderating effect of acceptance.

**Table 5:** Moderated multiple linear regression analysis showing the contribution of the interaction “mindfulness x acceptance” in predicting PCS-scores (*n* = 81).

Model	Variables	*B*	*SE (B)*	*β*	*R^2^*	*ΔR^2^*
1	Mindfulness (MAAS)	0.15	0.12	0.15	0.20**	
	Acceptance (AAQ-II)	−0.49	0.11	−0.50**		
2	Mindfulness x acceptance	0.00	0.12	0.00	0.20*	0.00

* *p*<0.05;

** *p*<0.001; Continuous variables were standardized.

## Discussion and Conclusions

In this study, we investigated the influence of mindfulness and acceptance on pain-related catastrophizing in patients with chronic pain. Based on our analysis, we conclude that general psychological acceptance is a strong predictor of pain-related catastrophizing, independent of gender, age or pain intensity. Patients with higher levels of acceptance catastrophize less about their pain complaints. Furthermore, we found that mindfulness was not related to pain-related catastrophizing. Even with the addition of acceptance as a moderator, mindfulness did not predict levels of pain-related catastrophizing in our sample of patient with chronic pain.

Our findings with regard to acceptance are consistent with the results of Chiros and O'Brien [22] and Viane et al. [23], who both found that higher levels of pain-related acceptance were related to lower levels of catastrophizing in participants with pain complaints. These authors defined acceptance as acceptance of chronic pain, while our study focused on general psychological acceptance. McCracken and Zhao-O'Brien [6] state that general psychological acceptance is broader than acceptance of pain. It includes acceptance of a variety of unwanted psychological experiences, not just pain. Based on our study, we can conclude that acceptance of psychological experiences outside of pain itself is related to catastrophizing about pain. Thus, an accepting attitude to unwanted experiences, whether they be pain or other psychological experiences, may prevent a person with pain from catastrophizing and may, according to the fear-avoidance model [24], [25], prevent the subsequent development of fear of pain, avoidance, hypervigilance, disuse, depression and disability.

The result that mindfulness was not related to pain-related catastrophizing is contrary to our expectations and to findings from previous research. For example, Schutze et al. [11] concluded that mindfulness was a unique predictor of catastrophizing in a sample of chronic pain patients from a multidisciplinary pain clinic. With respect to this finding we do have to take into account the way mindfulness is measured. In the present study we used the MAAS, which is a frequently used measure of mindfulness. However, inspection of the measure itself raises the question which aspect of mindfulness is measured with this questionnaire. The Mindful Attention Awareness Scale (MAAS) is described as a unidimensional measure [14], [15]. However, various authors state that mindfulness is a multifaceted construct. For example, Bishop et al. [26] propose a two-component model of mindfulness, with the components attention/awareness and acceptance. They describe mindfulness as self-focused attention characterized by openness and acceptance of experience. Baer et al. [27] also describe mindfulness as a multifaceted construct. They conducted a factor analysis of the combined pool of items from five mindfulness questionnaires and found that they contain five separate facets of mindfulness: 1) nonreactivity to inner experiences (*nonreact*), 2) observing/noticing/attending to sensations/perceptions/thoughts/feelings (*observe*), 3) acting with awareness/automatic pilot/concentration/nondistraction (*actaware*), 4) describing/labeling with words (*describe*), and 5) nonjudging of experience (*nonjudge*). These five elements of mindfulness were only modestly correlated with each other. With respect to the MAAS, Baer et al. [27] concluded that all items from the MAAS fell into the “actaware” category. Thus, the MAAS appears to measure only one facet of mindfulness, namely acting with awareness. In an exploratory factor analysis, McCracken and Thompson [28] did find the MAAS to consist of four separate factors. These factors (*acting with awareness, present focus, responsiveness*, and *social awareness*) do however all appear to encompass the aspect of “being in the present moment”, while the facets of mindfulness described by Baer et al. [27] provide a broader picture of mindfulness.

Based on their findings, Baer et al. [27] developed the Five Facet Mindfulness Questionnaire (FFMQ) as an alternative and multifaceted measure of mindfulness. In the study mentioned above, Schutze et al. [11] used the FFMQ as a measure of mindfulness. Further inspection of the results of their regression analysis shows that their finding that mindfulness was a unique predictor of catastrophizing applied particularly to the mindfulness facets of “nonreact” and “nonjudge”.

Based on the discussion by Baer et al. [27] into the facets of mindfulness, we can conclude that our result with regard to mindfulness may be limited to the mindfulness facet of “actaware”. Thus, although acting with awareness did not predict pain-related catastrophizing, other aspects of mindfulness might be related to catastrophizing.

The aim of the present study is exploratory. This is one of the first contributions to this subject and further study is needed. It is important to consider the limitations of the present study. The study is cross-sectional and therefore no causal inferences can be made. We only used self-report measures, which may be subject to various kinds of bias. Our sample comprises a high percentage of females of above average age, which is typical for chronic pain patients. Furthermore, our participants reported high levels of pain. Thus, our sample appears to consist of patients of typical age and sex with relatively severe pain complaints. Therefore, our results may not be generalizable to all pain sufferers. Also, our sample may have been heterogeneous with regard to, for example, location, duration and cause of the pain complaints. In future studies it would be interesting to test whether our results differ for groups with different types of pain complaints. Furthermore, the fact that all participants were included in the study based on their request for treatment may have led to bias. Treatment seeking may imply a certain degree of “unacceptance” of the pain. Thus, our sample may not be representative of all persons with pain, and therefore our data must be interpreted with caution.

Based on our study we conclude that general psychological acceptance is a strong predictor of pain-related catastrophizing and thus may play a role in the pain experience. It appears that the willingness to experience unwanted private events in order to pursue one's goals and values prevents a person from having an exaggerated negative orientation toward actual or anticipated pain experiences. This in turn could have a positive effect on the experienced pain intensity, disability and psychological distress [29]. Mindfulness, in the sense of acting with awareness, did not predict pain-related catastrophizing. However, a previous study by Schutze et al. [11] showed that mindfulness in the sense of non-reacting and non-judging did predict pain-related catastrophizing.

It appears that the results regarding mindfulness depend to a large extent on the definition used. Further research should reveal which facets of mindfulness are related to which pain-related constructs. Depending of the outcome of future studies, a critical stance toward the use of an “umbrella term” for various mindfulness facets may be needed. Indeed, if mindfulness proves to be a multifaceted construct in which the various facets are only modestly correlated to each other [27] and if these facets have widely different relations to pain-related constructs, should we call it all mindfulness or is a more nuanced description for the separate constructs needed? The theoretical debate into this should continue. For the present moment, based on the findings from the current study and from previous research, we recommend that the measurement of mindfulness should be broader than only measuring “acting with awareness”. In research and treatment, we recommend the use of a multifaceted questionnaire, for example the Five Facet Mindfulness Questionnaire (FFMQ) [27].

Furthermore, the findings from our study regarding the role of acceptance may have implications for the development of psychological treatment of patients with chronic pain. Based on our result that general psychological acceptance appears to play a role in the pain experience, we recommend the use of Acceptance and Commitment Therapy for chronic pain patients. It would be interesting to study whether the focus of ACT for chronic pain patients should be on acceptance of pain or on general psychological acceptance. Our results suggest that an accepting stance in life in general could have a positive influence on the pain experience and could make a positive contribution to other areas of life as well.
